# Supplementary material for: HDL Function and Size in Patients with On-Target LDL Plasma Levels and a First-Onset ACS
Source: Int J Mol Sci. 2023 Mar 11;24(6):5391. doi: 10.3390/ijms24065391 (PMC10048810; doi:10.3390/ijms24065391)
Supplement: Supplementary file 1 [file ijms-24-05391-s001.zip › ijms-2259333-supplementary.pdf]

Supplementary Material

HDL Function and Size in Patients with On-Target LDL Plasma Levels and a First-Onset ACS

Alberto Cordero [1,2,3,†], Natàlia Muñoz-García [4,†], Teresa Padró [3,4], Gemma Vilahur [3,4], Vicente Bertomeu-González [2,3], David Escribano [2,3], Emilio Flores [5], Pilar Zuazola [2] and Lina Badimon [3,4,6,*]

1 Cardiology Department, Hospital Universitario de San Juan, 03550 Alicante, Spain
2 Unidad de Investigación en Cardiología, Fundación Para el Fomento de la Investigación Sanitaria y Biomédica de la Comunitat Valenciana (FISABIO), 46020 Valencia, Spain
3 Centro de Investigación Biomédica en Red de Enfermedades Cardiovasculares (CIBERCV), 28029 Madrid, Spain
4 Cardiovascular-Program ICCC, Institut d'Investigació Biomèdica Sant Pau (IIB SANT PAU), 08041 Barcelona, Spain
5 Departamento de Análisis Clínicos, Hospital Universitario de San Juan, 03550 Alicante, Spain
6 Cardiovascular Research Chair, Autonomous University of Barcelona, 08193 Barcelona, Spain
* Correspondence: lbadimon@santpau.cat; Tel.:+34-935-565-882; Fax: +34-935-565-559
† These authors contributed equally to this work.

Table S1. Population characteristics of the healthy reference population and the patients admitted for chest pain

	**Reference Group** (*n*=31)	**Patients** (*n*=97)	***p-value***
Age (years, mean ± SEM)	53.8±0.8	64.9±1.2	**<0.001**
Male/Female,*n*	16/15	72/25	**0.018**
Weight (Kg)	87.0±2.4	83.0±1.8	0.246
BMI (Kg/m^2, mean ± SEM)	30.6±0.5	28.6±0.5	**0.020**
**Clinical Data (mean ± SEM)**			
Total Cholesterol (mg/dL)	195.4±5.5	172.2±3.9	**0.003**
Triglycerides (mg/dL)	120.2±13.0	146.1±8.8	0.135
LDL-C (mg/dL)	121.9±4.7	100.1±3.6	**0.002**
HDL-C (mg/dL)	49.5±1.9	44.5±1.4	0.068
Non-HDL-C (mg/dL)	145.8±5.0	127.7±4.0	**0.018**
ApoA-I (mg/ml)	1.7±0.1	1.5±0.1	0.066
HDL/LDL	0.42±0.02	0.50±0.03	0.087
TG/HDL	2.7±0.4	3.8±0.3	0.088
Creatinine (mg/dL)	0.79±0.02	0.94±0.03	**0.006**
Fasting Plasma Glucose (mg/dL)	90.2±1.7	122.8±5.7	**0.002**
GOT (UI/l)	18.5±0.8	46.3±5.6	**0.005**
GPT (UI/l)	20.4±2.2	33.1±2.6	**0.025**
Urea (mg/dL)	15.0±0.6	39.0±1.6	**<0.001**

Results are expressed as Mean ± SEM. Student's t-test for unpaired samples was applied for two group comparisons. Only *p*-values <0.05 are considered significant. Abbreviations: BMI= Body Mass Index, HDL= High Density Lipoprotein, LDL= Low Density Lipoprotein, ApoA-I= Apolipoprotein A-I, GOT=Glutamic-oxaloacetic Transaminase, GPT= Glutamic-pyruvic Transaminase.

Table S2. Differences in the particle numbers of each lipoprotein class (expressed in percentage) in the chest pain groups

		**ACS** (*n*=69)		
	**Non-ACS** (*n*=25)	**NSTEMI** (*n*=30)	**STEMI** (*n*=39)	***p-value***
**VLDL particle number (%)**				
Small VLDL-P	87.7±0.6	86.9±0.4	86.1±0.5*	0.077
Medium VLDL-P	9.8±0.6	10.6±0.4	11.4±0.5*	0.062
Large VLDL-P	2.5±0.1	2.5±0.1	2.5±0.1	0.638
**LDL particle number (%)**				
Small LDL-P	56.2±1.5	56.6±0.9	56.2±0.8	0.954
Medium LDL-P	28.2±1.3	27.1±0.7	27.7±0.6	0.676
Large LDL-P	15.6±0.3	16.3±0.3	16.1±0.3	0.231
**HDL particle number (%)**				
Small HDL-P	62.2±1.2	56.7±1.3*	56.3±1.2*	**0.004**
Medium HDL-P	36.7±1.2	42.1±1.3*	42.4±1.2*	**0.004**
Large HDL-P	1.1±0.05	1.2±0.05	1.3±0.04*	**0.012**

Results are expressed as Mean ± SEM. ANOVA test was applied for multiple comparisons, Bonferroni post-hoc was run for two group comparisons after ANOVA: *, significant changes versus Non-ACS group. Only *p*-values <0.05 are considered significant. Abbreviations: ACS= Acute Coronary Syndrome, NSTEMI= non-ST segment elevation-myocardial infarction, STEMI= ST-segment elevation-myocardial infarction, VLDL= Very Low Density Lipoprotein, LDL= Low Density Lipoprotein, HDL= High Density Lipoprotein.

Table S3. Lipoprotein particle number and size in all groups

	Reference Group (*n*=31)	Non-ACS (*n*=25)	ACS (*n*=69) NSTEMI (*n*=30)	ACS (*n*=69) STEMI (*n*=39)	*p-value*
**Triglyceride content in lipoprotein particles (mg/dL)**					
VLDL-TG	75.6±8.5	85.6±6.9	77.2±5.6	87.1±6.5	0.550
IDL-TG	10.4±0.5	11.4±0.3	11.2±0.5	12.1±0.6*	0.124
LDL-TG	14.0±0.6	14.1±0.9	13.4±0.7	15.0±0.7	0.453
HDL-TG	12.5±0.7	13.9±0.5	12.2±0.5	12.3±0.7	0.294
**VLDL particle number (nmol/L)**					
Small VLDL-P	44.7±4.3	54.7±4.7	48.9±3.8	54.8±4.3	0.279
Medium VLDL-P	6.4±1.1	5.7±0.4	5.6±0.3	6.8±0.4	0.534
Large VLDL-P	1.3±0.1	1.5±0.1	1.4±0.1	1.5±0.1	0.274
Total VLDL-P	52.4±5.3	61.9±5.1	55.9±4.2	63.1±4.7	0.349
**LDL particle number (nmol/L)**					
Small LDL-P	713.1±21.5	691.9±28.6	642.8±19.6*	677.5±24.7	0.222
Medium LDL-P	409.0±19.7	367.3±32.4	312.8±17.1*	336.7±14.9*	**0.008**
Large LDL-P	212.8±5.5	194.3±9.9*	184.9±4.8*	191.3±4.9*	**0.013**
Total LDL-P	1335.0±34.7	1253.5±62.7	1140.5±35.2*	1205.5±40.6*	**0.016**
**HDL particle number (nmol/L)**					
Small HDL-P	18.2±0.5	18.3±0.8	14.8±0.7*ǂ	13.8±0.6*ǂ	**<0.001**
Medium HDL-P	11.0±0.3	10.6±0.4	10.7±0.3	10.0±0.2*	0.102
Large HDL-P	0.31±0.01	0.31±0.01	0.30±0.01	0.30±0.01	0.548
Total HDL-P	29.5±0.7	29.2±1.0	25.8±0.8*ǂ	24.1±0.6*ǂ	**<0.001**
**Average particle diameter (nm)**					
VLDL	42.2±0.05	42.1±0.05*	42.1±0.04	42.2±0.04ǂ	0.112
LDL	21.1±0.06	21.0±0.06	21.0±0.04	21.0±0.04	0.407
HDL	8.3±0.01	8.3±0.02	8.4±0.02*ǂ	8.4±0.02*ǂ	**<0.001**

Results are expressed as Mean ± SEM. ANOVA test was applied for multiple comparisons, Bonferroni post-hoc was run for two group comparisons after ANOVA: *, significant changes versus Reference group and; ǂ, significant changes of NSTEMI/STEMI groups vs Non-ACS. Only *p*-values <0.05 are considered significant. Abbreviations: ACS= Acute Coronary Syndrome, NSTEMI= non-ST segment elevation-myocardial infarction, STEMI= ST-segment elevation-myocardial infarction, VLDL= Very Low Density Lipoprotein, IDL= Intermediate Density Lipoprotein, HDL= High Density Lipoprotein, TG= Triglycerides.

Table S4. Differences in the particle numbers of each lipoprotein class (expressed in percentage) in all groups

	Reference Group (n=31)	Non-ACS (n=25)	ACS (n=69) NSTEMI (n=30)	STEMI (n=39)	*p-value*
**VLDL particle number (%)**					
Small VLDL-P	86.0±0.6	87.7±0.6*	86.9±0.4	86.1±0.5ɫ	0.099
Medium VLDL-P	11.4±0.6	9.8±0.6*	10.6±0.4	11.4±0.5ɫ	0.099
Large VLDL-P	2.5±0.1	2.5±0.1	2.5±0.1	2.5±0.1	0.607
**LDL particle number (%)**					
Small LDL-P	53.7±1.2	56.2±1.5	56.6±0.9	56.2±0.8	0.202
Medium LDL-P	30.3±1.1	28.2±1.3	27.1±0.7*	27.7±0.6*	0.077
Large LDL-P	16.0±0.3	15.6±0.3	16.3±0.3	16.1±0.3	0.398
**HDL particle number (%)**					
Small HDL-P	61.6±0.7	62.2±1.2	56.7±1.3*ɫ	56.3±1.2*ɫ	**<0.001**
Medium HDL-P	37.4±0.7	36.7±1.2	42.1±1.3*ɫ	42.4±1.2*ɫ	**<0.001**
Large HDL-P	1.1±0.02	1.1±0.05	1.2±0.05*	1.3±0.04*ɫ	**<0.001**

Results are expressed as Mean ± SEM. ANOVA test was applied for multiple comparisons, Bonferroni post-hoc was run for two group comparisons after ANOVA: *, significant changes versus Reference group and; ɫ, significant changes of NSTEMI/STEMI groups vs Non-ACS. Only p-values <0.05 are considered significant. Abbreviations: ACS= Acute Coronary Syndrome, NSTEMI= non-ST segment elevation-myocardial infarction, STEMI= ST-segment elevation-myocardial infarction, VLDL= Very Low Density Lipoprotein, LDL= Low Density Lipoprotein, HDL= High Density Lipoprotein.

Table S5. Effect of the blood collection time on the studied variables

Group:<br>ACS (*n*=70)	Blood Collection<br>**Within the 24h post-event**<br>(*n*=18)	 **During the 48-72h post-event**<br>(*n*=29)	 **After 72h post-event**<br>(*n*=22)	***P-value***
Lipid parameters (mg/dL)				
Total Cholesterol	173.8±7.1	172.1±6.7	154.5±7.0	0.116
LDL-C	96.6±7.0	104.4±6.3	91.3±5.3	0.304
HDL-C	47.9±3.7	40.5±2.0*	37.4±1.8*	**0.019**
ApoA-I	1.59±0.1	1.40±0.1	1.30±0.1*	**0.026**
Triglycerides	145.6±21.1	145.3±15.4	150.6±25.2	0.978
HDL functionality				
TRAP (% HDL inhibition capacity)	69.9±2.9	67.8±2.5	69.4±2.6	0.807
HDL baseline oxidation (RFU)	5.6±0.6	4.8±0.3	5.5±0.4	0.380
Cholesterol Efflux (%)	19.7±1.3	19.3±1.0	19.5±1.0	0.969
Biochemistry				
GOT(UI/l)	67.4±18.2	62.4±13.1	35.9±3.4	0.205
GPT(UI/l)	34.1±6.3	39.4±6.0	35.1±3.9	0.766

Results are expressed as Mean ± SEM. ANOVA test was applied for multiple comparisons, Bonferroni post-hoc was run for two group comparisons after ANOVA: *, significant changes versus "within 24h post-event". Only *p*-values <0.05 are considered significant. Abbreviations: ACS= Acute Coronary Syndrome, LDL= Low Density Lipoprotein, HDL= High Density Lipoprotein, ApoA-I= Apolipoprotein A-I, TRAP= The total Radical-trapping Antioxidative Ppotential, GOT=Glutamic-oxaloacetic Transaminase, GPT= Glutamic-pyruvic Transaminase.

Figure S1. Cholesterol efflux capacity (CEC [%]) vs Basal HDL oxidative grade (AFU)

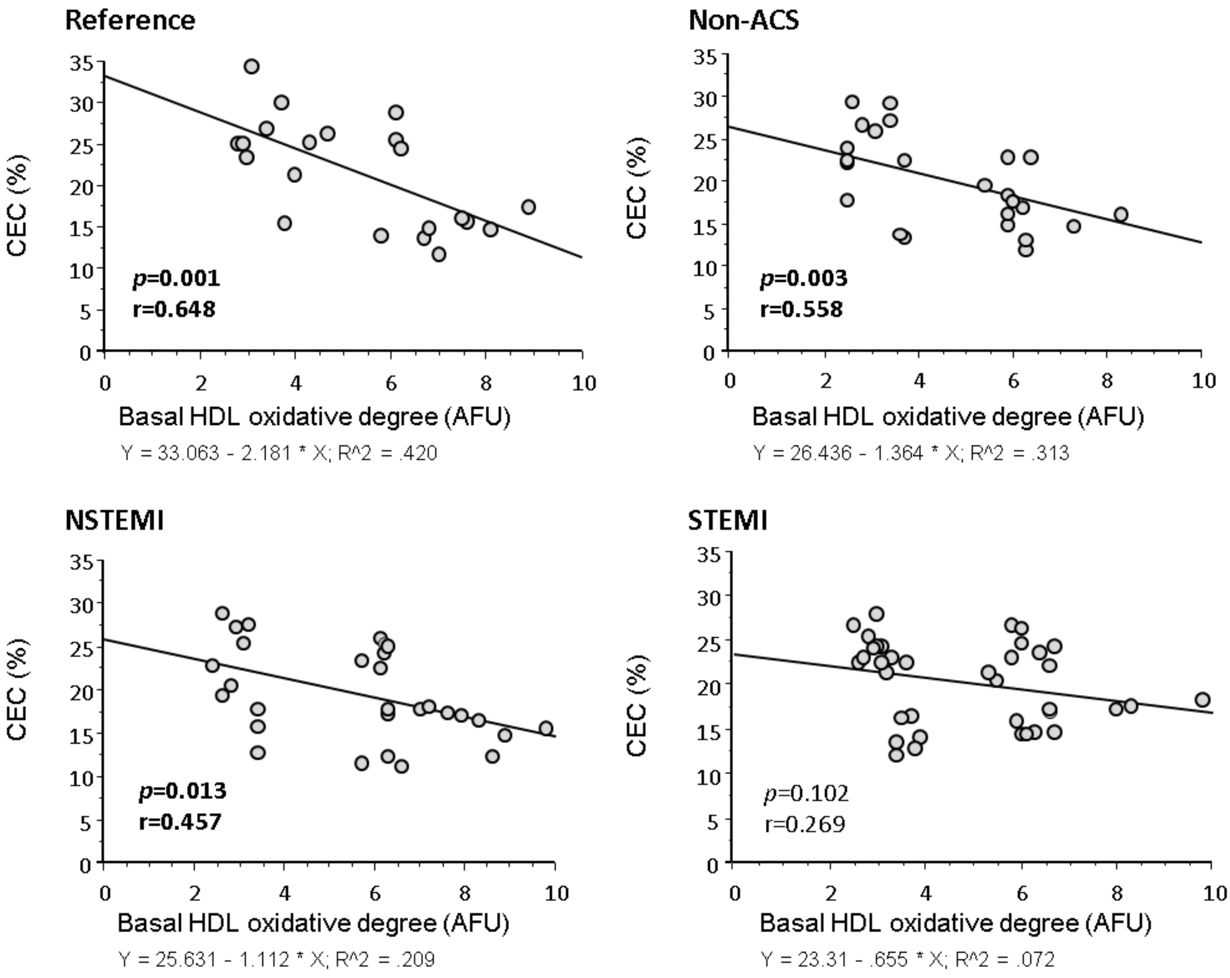

Figure S1. Bivariate scatergrams. Cholesterol efflux capacity (CEC [%]) vs Basal HDL oxidative grade (AFU) in healthy subjects (Reference), Non-ACS, NSTEMI and STEMI patients. Correlation and significance values were obtained using the Pearson test. Only *p*-values <0.05 are considered significant.

Figure S2. Cholesterol efflux capacity (CEC [%]) vs HDL oxidative grade (AFU)

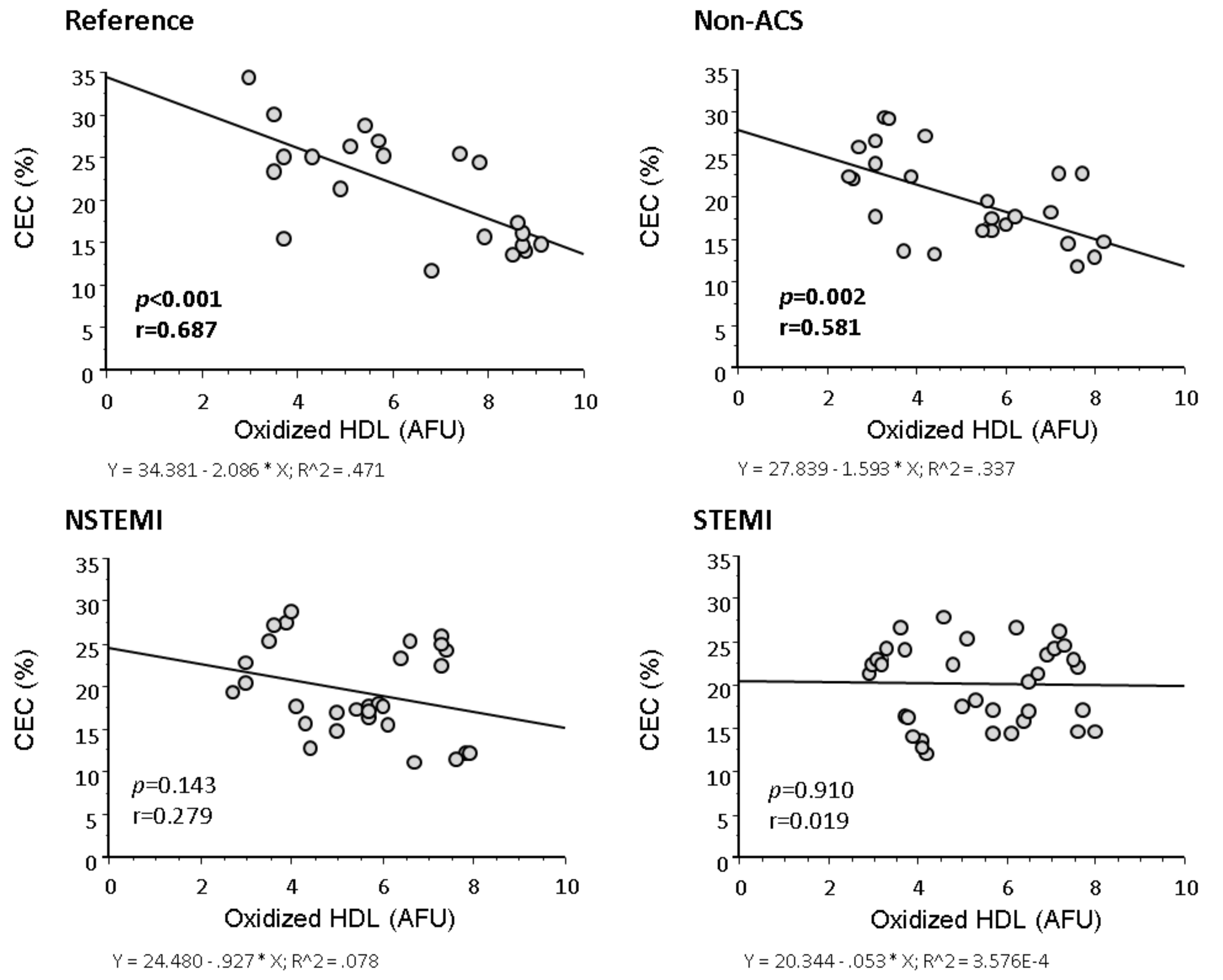

Figure S2. Bivariate scatergrams. Cholesterol efflux capacity (CEC [%]) vs HDL oxidative grade (AFU) in healthy subjects (Reference), Non-ACS, NSTEMI and STEMI patients. Correlation and significance values were obtained using the Pearson test. Only *p*-values <0.05 are considered significant.
